# Clinical studies using stem cells for treatment of retinal diseases: state of the art

**DOI:** 10.5935/0004-2749.20200037

**Published:** 2020

**Authors:** Carina Costa Cotrim, Rodrigo Jorge, Maria Carolina de Oliveira, Fabiano Pieroni, André M. Vieira Messias, Rubens Camargo Siqueira

**Affiliations:** 1 Department of Ophthalmology, Otorhinolaryngology and Head and Neck Surgery, Faculty of Medicine of Ribeirão Preto, Universidade de São Paulo, Ribeirão Preto, SP, Brazil; 2 Department of Internal Medicine Faculty of Medicine of Ribeirão Preto, Universidade de São Paulo, Ribeirão Preto, SP, Brazil; 3 Department of Internal Medicine , Faculty of Medicine of Ribeirão Preto, Universidade de São Paulo, Ribeirão Preto, SP, Brazil; 4 Faculty of Medicine, Universidade Estadual de São José do Rio Preto, São José do Rio Preto, SP, Brazil

**Keywords:** Stem cells, Retinal progenitor cells, Embryonic stem cells, Induced pluripotent stem cells, Bone marrow-derived stem cells, Células-tronco, Células progenitoras da retina, Células-tronco embrionárias, Células-tronco pluripotentes ind uzidas, Células-tronco derivadas da medula óssea

## Abstract

Degenerative retinal diseases such as retinitis pigmentosa, Stargardt’s macular dystrophy, and age-related macular degeneration are characterized by irreversible loss of vision due to direct or indirect photoreceptor damage. No effective treatments exist, but stem cell studies have shown promising results. Our aim with this review was to describe the types of stem cells that are under study, their effects, and the main clinical trials involving them.

## Abstract (pt)

As doenças degenerativas da retina, como retinose pigmentar, distrofia macular de Stargardt e degeneração macular relaciona à idade, são caracterizadas por perda irre versível da visão devido a danos diretos ou indiretos aos fotorreceptores. Não existem tratamentos eficazes, porém os estudos com células-tronco mostraram resultados promissores. Nosso objetivo com esta revisão foi descrever os tipos de células-tronco em estudo, seus efeitos e os principais ensaios clínicos que as envolvem.

## INTRODUCTION

Stem cells are undifferentiated immature cells without complex structures that can differentiate into other types of body cells^(1)^. They are characterized by three general properties: unlimited self-renewal ability, a nonspecialized status, and the ability to differentiate into various cell types. Stem cells can be classified according to various criteria. A broad classification regards their differentiation potential: multipotent stem cells can differentiate into a limited number of cell types, whereas pluripotent stem cells can differentiate into any cell type found in the adult body. In turn, multipotent stem cells may be subclassified according to their origin: fetal stem cells are derived from a variety of developing fetal tissues and adult stem cells from adult functional tissues; also, neural stem cells are derived from neuroectodermal lines and non-neural stem cells from neuroectodermal lines.

The cornea was the first ocular segment tested with therapies involving stem cells owing to their ease of access, and it was later followed by studies on the retina, which has a more complex structure, is vascularized, and exhibits a wider variety of cells^(2)^. Although access to the retina is more complex than access to the cornea, the eye in general displays an immunological tolerance that can reduce the rejection of transplanted cells. In addition, the numerous tools available for measuring ocular structures and functions such as optical coherence tomography (OCT), angiofluoresceinography, and multifocal electroretinography (ERG) allow for incomparable detections of structure-function correlations. Thus, cell therapy clinical assays have been prioritized for retinal disorders^(3)^.

Despite the great investment in research on stem cells, increasing numbers of clinics offer treatments with stem cells without evidence for the safety or efficacy of the procedures. This phenomenon, known as “stem cell tourism”, is in frank expansion, and important adverse effects have been reported in the treatment of various systemic diseases. Thus, a rigorous conduct code should be applied to research on cell therapy, obeying the guidelines for good clinical practice^(4)^.

## METHODS

We conducted a review on the main clinical studies dealing with the use of stem cells for age-related macular degeneration (AMD), retinitis pigmentosa (RP), and Stargardt’s macular dystrophy. We selected studies on the basis of advanced PubMed searches using the following key words: “Stem cells and AMD”, “Stem cells and Retinitis Pigmentosa”, “Stem cells and retinal dystrophy”, and “Stem cells and Stargardt”. We also included data obtained from the clinicaltrials.gov site, which presents ongoing and unpublished research. We considered all studies found that were related to the research objective.

### Mechanism of action of stem cells

Cell therapy is expected to produce two different main effects on the retina: a) a regenerative effect or cell replacement therapy and b) a trophic or functional rescue therapy^(5)^.

Regenerative therapy consists of the replacement of damaged tissue with new cells that can provide functional capacity. The main studies based on this type of therapy use cultures of retinal pigmented epithelial cells (RPE) from embryonic stem cells (ESC) injected into the subretinal space. Because of the heterologous origin of the cells, these patients require immunosuppression after the therapy^(3,6-8)^.

Trophic therapy is another form of cell therapy employing stem cells. The cells used are already adult and do not have the same potential for transformation as ESCs. Bone marrow, umbilical cord, and fat tissues can be used as potential sources of adult cells. The advantage regarding the acquisition of these cells, in addition to the absence of ethical questions that still involve the use of ESCs, is their ease of acquisition. Although they cannot differentiate into retinal cells, they release cytokines and neurotrophic and apoptosis-inhibiting factors that can rescue altered cells creating a microenvironment favorable to the avoidance of cell death and stalling degeneration progression. This effect is a paracrine event (cell signaling in which the target cell is close to the cell that releases the signal) and leads to angiogenesis, inflammation reduction, antiapoptotic effects, remodeling of the extracellular matrix, and activation of neighboring stem cells^(9)^.

### Routes of cell administration

The routes tested for cell therapy in retinal diseases include systemic (intravenous) administration, intravitreous (IVT) injections, suprachoroid space injections, and subretinal injections. The two techniques for subretinal injection are a) injection of cell suspensions and b) injection of cells adhered to a scaffold^(8)^.

At present, procedures based on the use of scaffolds require a larger retinotomy for cell delivery to the subretinal space than does the delivery of disperse cells. As a result, the delivery of cells adhered to scaffolds may involve increased risks of cell migration to the vitreal cavity, where the cells may undergo transdifferentiation or uncontrolled proliferations, formation of scar tissues (depending on the cell types), and development of epiretinal scar tissues and/or retinal detachments during the postoperative period. In addition, the scaffolds may migrate from the site where they were placed intraoperatively to sites distant from the fovea. If postoperative migration occurs, the trophic effects of the transplant may be reduced and the replacement effects may be completely lost. The intraoperative use of devices such as fluids heavier than water may fix the scaffold in the appropriate site at the time of reinsertion of the retina (after delivery of the cells to the subretinal space), which may mitigate the risk of scaffold migration^(10)^.

In contrast to IVT injections, cell transfers to the subretinal space require localized retinal detachment. If the type of therapeutic cells functions with a rescue mechanism involving the production and diffusion of trophic factors, then avoiding foveal detachment may be avoided by positioning the cells in an extrafoveal site.

Two major approaches are used to inject cells into the subretinal space: 1) injections of RPE cell suspensions and 2) grafts of RPE cell monolayers seeded onto support membranes (scaffold). In contrast to RPE suspensions, cells on patches are delivered fully differentiated, polarized, and with formed tight junction barriers, that is, in a form close to their native configurations^(11)^. Although some investigators have reported that cell suspensions do not get properly integrated with damaged areas, they also result in marked visual function improvements. It is important to emphasize that this approach is relatively easy and has been employed in recent clinical trials^(12)^.

### Cell types

Pluripotent cells have the ability to form all types of embryonic tissues (ectoderm, mesoderm, and endoderm). The type identified and studied for the longest time is the ESC, followed by induced pluripotent stem cells (iPSCs).

**Figure 1:**
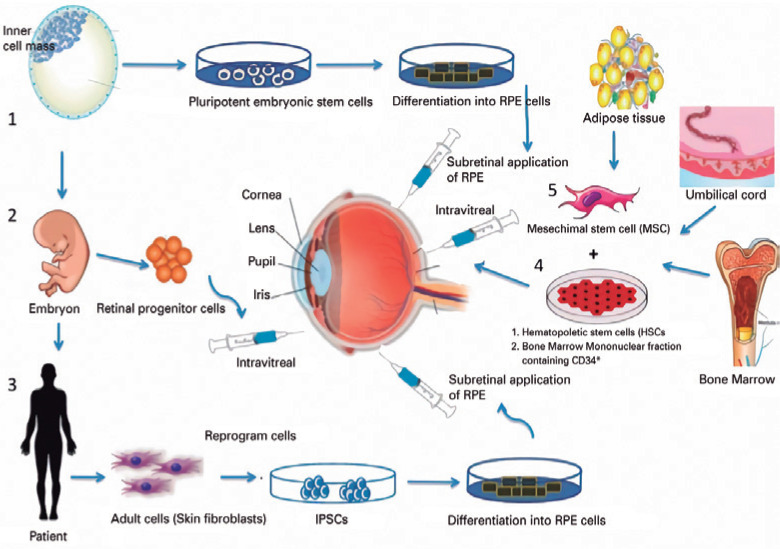
Schematic illustration off the various types of stem cells. 1, Embryonic stem cell; 2, retinal progenitor cells; 3, induced pluripotent stem cells (iPSCs); 4, bone marrow-derived hematopoietic stem cells. RPE (retinal pigment epithelium); 5, mesenchymal stem cell (MSC). Adipose tissue, umbilical cord, and bone marrow may originate MSC and HSCs. The main studies involving MSC are preclinical.

Among the different types of multipotent stem cells identified, the following have been most frequently assessed for potential treatment of degenerative retinal diseases: 1) fetal stem cells from neural lines including fetal retinal progenitor cells (fRPCs) and fetal cortical progenitor cells (fCPCs); 2) adult stem cells from neural lines including ciliary epithelium-derived stem cells (CESCs), RPE stem cells, and Muller glial cells (MCs); 3) adult stem cells from non-neural lines including those derived from umbilical tissues (UTSCs) and those derived from bone marrow (BMDSCs). Pluripotent stem cells, on the other hand, include ESCs and iPSCs^(13)^ (Figure 1). Among the described stem cells, those most frequently used in clinical studies are listed below.

### Embryonic stem cells (ESCs)

ESCs are derived from the internal cell mass of embryos in the form of blastocysts and possess abilities for self-renewal and for differentiation into all types of adult cells derived from any of the three germinative layers. After the blastocyst period (4 to 5 days after fecundation) the embryonic cells become differentiated into those of organs such as heart and nerve cells and can no longer be considered to be stem cells. Studies have shown that ESCs can differentiate into photoreceptor progenitor cells, photoreceptors, or RPE in rats and in humans^(14-16)^. However, their unlimited ability to multiply also leads to problems such as the formation of teratomas and other tumors and exacerbated immune reactions^(17,18)^. ESCs have been associated with the formation of teratomas^(19)^. *In vitro* cell differentiation theoretically resolves the problem of cell multiplication in a disorganized manner and the formation of intraocular tumors. But, although ESCs are promising for retinal cell replacement therapies, ethical and immune questions such as rejection persist and must be considered. According to Thomson et al.^(20)^, ESCs can be generated from a single cell obtained from embryos created for reproductive purposes based on *in vitro* fertilization without sacrificing the embryo^(18)^ (a strategy that would reduce the ethical implications of the therapy (see Table 1).

**Table 1:** Sources of Cells for Retinal Cell Therapy

Cell type	Source (examples)	Advantages	Disadvantages	Studies
Embryonic stem cells (ESC)	Derived from the internal cell mass of embryos	Pluripotent Differentiation into all types of adult cells	Ethical and immune questions Formation of tumors	**Subretinal applications of RPE derived from embryonic cells:** *Astellas Institute for Regenerative Medicine, USA,* Schwartz et al^(21,22)^, (NCT02941991/NCT02445612)^(25,26)^ *Regenerative Patch Technologies, USA, clinical trial* (NCT02590692)^(27)^. Southwest Hospital, China, NCT02749734)^(28)^ *The First Affiliated Hospital of Zhengzhou University,* China, (NCT03046407)^(29)^. Universidade Federal de São Paulo (Unifesp), Brazil, (NCT02903576)^(30)^.
Induced pluripotent stem cells (iPSCs)	Somatic cells that are terminally differentiated into skin tissues (fibroblasts), genetically reprogrammed by retroviral transduction.	Pluripotent Absence of ethical problems Low need to use immunosuppressive drugs	Higher cost Mutations in the cellular reprogramming process	**Subretinal application of RPE derived from iPSCs:** Takahashi, R1KEN Institute, Japan. ^(31,38,39)^
Bone marrow-derived hematopoietic stem cells (BMDSC)	Hematopoietic stem cells (HSCs) from the bone marrow of adults (femur, ribs, and sternum), umbilical cords, and the placenta.	Multipotent No need for systemic immunosuppression	These cells lack the same potential for transformation in other cells than ESCs	**Intravitreous Injection:** *Department of Ophthalmology and Vision Science, University of California-Davis Eye Center, EUA,* Park *et* al.^(45)^, (NCT01736059) Faculdade de Medicina de Ribeirão Preto, ( USP), Brazil, Siqueira et al.^(7,8,46,47)^ (NCT01068561/ NCT0151812 7/ NCT01518842
Retinal progenitor cells (RPC)	Population of immature cells responsible for the production of retinal cells during embryonic development; acquired from ocular tissues of aborted 12-to-16-week fetuses	Multipotent	These cells lack the same potential for transformation in other cells than ESCs	**Subretinal application:** Liu et al.^(46)^ **Intravitreous injection:** jCyte e o *California Institute for Regenerative Medicine* (C1RM)^(49)^, Barruch Kuppermann^(48)^, (CT03073733)

### Use of ESCs

Schwartz et al.^(21)^ described a phase I/II clinical study with RPE derived from ESCs for the first time in two patients with advanced stages of Stargardt’s disease and AMD. RPE cells were injected into the subretinal space in a pericentral region by pars plana vitrectomy. The patients required the use of immunosuppression after therapy because of the heterologous origin of the cells^(3,6-8)^ but showed significantly improved vision, with no adverse effects. Schwartz et al.^(22)^ reported the results of 22 months of follow-up of 18 patients, nine of them with AMD and nine with Stargardt’s disease. The visions improved in 10 patients, remained stable in seven patients, and worsened in only one patient. The contralateral eyes, used as controls, did not show vision improvements. After 4 years of the same study, Schwartz et al.^(23)^ calculated a visual acuity (VA) improvement of up to 15 letters within 6 months. In 72% of the cases, increased pigmentation in the margins of macular atrophy was consistent with RPE transplants. Side effects included endophthalmitis (one case), cataracts (four cases), and effects related to immunosuppression such as urinary tract infections and gastroenteritis. Two cases of skin melanoma were also associated with the treatment, but they found no growths of intraocular tumors. Mehat et al.^(24)^ reported a 12-month follow-up of 12 patients with Stargardt-type degenerations who underwent subretinal injection of RPE cells. They found no side effects associated with rejection (inflammation or uncontrolled cell growth). All patients received an immunosuppressor and showed effects such as lethargy, and one patient experienced reactivation of a herpes virus infection. All 12 patients developed subretinal hyperpigmentation within the injected areas, which were shown to be dosede pendent and in many cases were associated with a hyperreflective signal in the OCT, suggesting the survival of RPE cells from donor-derived hESCs. However, no benefits regarding vision, microperimetry, or quality of life were found either. The authors pointed out that, the advance disease of their patients probably meant that a longer period of observation would have been necessary to observe other results. The authors also emphasized that a thinning of the retina above the injected area obser ved in some patients alerted to the need for caution about the use of this procedure in patients with less advanced disease. Da Cruz et al.^(11)^ reported the results of 12 months of follow-up of two patients with severe exudative ARMD who underwent subretinal implants of hESC-RPE patches. The authors used a human-vitronectin-coated polyester membrane not previously reported for this use. They employed local immunosuppression with fluocinolone and observed a gain in VA of more than 15 letters at the end of 12 months. Despite these favorable results, severe complications such as exposure of the fluocinolone suture, retinal detachment, and worsening of diabetes due to the use of a systemic corticoid also ensued. Importantly, these complications were successfully resolved.

Some investigations on the subretinal application of RPE-derived embryonic cells are ongoing. These studies use scaling of the injected cells. The main research group, coordinated by the Astellas Institute for Regenerative Medicine, the same company responsible for the studies of Schwartz et al.^(21,22)^, expects to complete the studies in 2019 and is working on Stargardt’s dystrophy (Registration in the clinical trial NCT02941991/ NCT02445612)^(25,26)^. Another US group, Regenerative Patch Technologies, is investigating the procedure for dry AMD (NCT02590692)^(27)^. A study on 15 patients with AMD and Stargardt’s disease is being conducted in China at the Southwest Hospital (NCT02749734)^(28)^. Also in China, another study at The First Affiliated Hospital of Zhengzhou University is in the phase of recruitment of patients with dry AMD (NCT03046407)^(29)^. Finally, in Brazil, patients with dry and exudative AMD and Stargardt’s disease have been included in a study by the Universidade Federal de São Paulo (Unifesp) (NCT02903576)^(30)^.

### Induced pluripotent stem cells (iPSCs)

iPSCs are derived from adult tissues and were first described by Takahashi and Yamanaka^(31)^. They are somatic cells terminally differentiated as skin tissues (fibroblasts), and they are genetically reprogrammed by retroviral transduction that confers to them properties like those of ESC. They are typically obtained after the introduction of products of specific gene sets associated with pluripotency or “reprogramming factors” to a given cell type. The original set of reprogramming factors (also called Yamanaka factors) consists of the transcription factors Oct4 (Pou5f1), Sox2, cMyc, and Klf4. Although this combination is more conventional for the production of iPSCs, each of these factors can be functionally replaced with related transcription factors, miRNAs, small molecules, or even unrelated genes that are lineage specifiers^(13)^. Since they are adult cells of the patient himself (autologous), compared with ESCs, they have advantages such as a reduced need to use immunosuppressive drugs to avoid rejection and the absence of ethical problems since they are not derived from embryos. However, they also have a greater chance of forming tumors^(32)^. Cellular aberrations may occur because of the cellular stress that accompanies reprogramming^(33)^. Thus, producing RPE cells from iPSCs is possible using embryonic cells^(34)^. iPSC-derived RPE cells form monolayers with tight junctions expressing genes necessary for vision can perform phagocytosis^(34,35)^ (Table 1).

### Use of iPSCs

On the basis of the good results of preclinical studies, such as those on the use of subretinal iPSC-derived RPE in mice demonstrated by Li et al.^(36,37)^, in 2014, Masayo Takahashi (RIKEN, Japan) conducted the first clinical trial using iPCS. The major objective was to assess safety, viability, and side effects. However, the study was interrupted in March 2015 after demonstrating mutations in the iPSCs in the second patient scheduled to receive the cells. These mutations were not detectable in the original fibroblasts of the patient, but whether the process of induction and reprogramming induced abnormalities in the iPSCs is unclear. In addition, changes in the laws of regenerative medicine in Japan have hampered the continuity of the studies ^(38,39)^.

### Bone marrow-derived hematopoietic stem cells

BMDSCs are multipotent stem cells that give origin to all types of blood cells, including the myeloid lineages (monocytes, macrophages, neutrophils, basophils, eosinophils, megakaryocytes, erythrocytes, platelets, and dendritic cells) and lymphoid lineages (T and B cells and natural killer cells). The expression of CD34 is the hallmark of these cells. Hematopoietic stem cells are found in the bone marrow of adults and can be directly obtained from the hip (iliac crest), the site most frequently used for collection, and also from other bones such as the femur, the ribs, and the sternum by puncture with a special needle and syringe by the hematology team. The cells can also be obtained from blood after pretreatment with cytokines such as granulocyte colony-stimulating factors, which induce the cells to mobilize from the bone marrow compartment to the bloodstream. Other sources of these cells for clinical and scientific use include the umbilical cord and the placenta^(8,9)^. The advantage of using these cells in relation to ESCs and to induced cells (IPCS) is the absence of tumor induction, whereas the disadvantage is the lower ability of these cells to differentiate into retinal cells because of their paracrine effects^(8,9)^ (Table 1).

### Use of BMDSCs

An alternative approach to stem cell therapy is the induction of functional rescue of affected cells in the retina by the introduction of stem cells that promote a paracrine trophic effect. This treatment, which is possible with the use of BMDSC^(40)^, is not specific for a given disease and can have broad clinical applications. Administering these cells by IVT injection without adverse effects is highly desirable.

In contrast to vitrectomy surgery for subretinal cell administration, which requires hospitalization and a significant time of postoperative recovery, IVT cell injections can be performed on an outpatient basis, with minimal recovery time. Finally, as BMDSCs are usually obtained from adult tissues, their use elicits no ethical questions. For autologous use, no systemic immunosuppression is needed. These adult stem cells are multipotent and have a more limited ability to differentiate and divide than embryonic and iPSCs. These characteristics of adult BMDSCs may cause them to be less ideal for regenerative therapy than other cell types for tissue replacement^(41)^. However, the same characteristics render adult stem cells safer than others in clinical trials because they involve a minimal risk of teratoma.

Many investigators have assessed the safety and efficacy of the use of these cells for retinal diseases. In contrast to embryonic cells, BMDSCs are administered by IVT injections, a simple procedure routinely used in the clinical practice for other treatments.

In Brazil, clinical studies with these cells started in 2009 at the Faculty of Medicine of Ribeirão Preto, in the University of São Paulo, for the treatment of RP (ClinicalTrials.gov, NCT01068561), macular degeneration and Stargardt (NCT01518127), and ischemic retinopathy including diabetic retinopathy (NCT01518842). Siqueira^(8)^ carried out a phase I non-randomized prospective study on patients with RP. IVT injection of BMDSCs in the eyes of patients with RP was not associated with any detectable structural or functional toxicity over a period of 10 months. In another study on patients with RP (phase II), Siqueira et al.^(42)^ published the response of one of the participants, who showed improvement of cystoid edema, of VA, and of microperimetry sensitivity after BMDSC injection. Siqueira et al.^(9)^ analyzed a quality of life questionnaire (VFQ-25) administered to 20 patients with RP submitted to BMDSC injection and observed an increased quality of life during the third month of follow-up with later deterioration. In the same year, Siqueira et al.^(43)^ conducted another study with IVT injected stem cells in two patients with macular ischemia due to diabetic retinopathy in one of them and to occlusion of the central retinal vein in the other. Both patients showed improvement of VA, microperimetry, multifocal electroretinogram (ERGmf), and retinal edema as assessed by OCT. Cotrim et al.^(44)^ reported the results of intravitreous use of BMDSC in 10 patients with the dry form of AMD, with improved VA during the 12-month follow-up and improved microperimetry and quality of life (VFQ-25) at 6 months.

A California group (Department of Ophthalmology and Vision Science, University of California-Davis Eye Center [USA]) obtained results similar to those reported in Brazil with the use of BMDSCs. In a phase I study on six patients (six eyes) with retinal ischemia or degeneration, Park et al.^(45)^ observed no local or systemic side effects. They also detected improved VA in six patients with Stargardt’s disease, AMD, occlusion of the central artery, and vein of the retina, and RP. Improvements ranged from 3 to 65 letters.

Adipose tissue-derived “stem cells” (ATDSC) are also for autologous use, but the results of studies using them have been different from those presented above. A series of three patients with severe visual loss after the IVT injection of ATDSC was described in the USA^(44)^. The patients’ severe visual losses after the injection were associated with ocular hypertension, hemorrhagic retinopathy, vitreous hemorrhage, combined traction, and rhegmatogenous retinal detachment, or lens dislocation. The authors speculated that the ATDSCs administered by IV injection may transform into myofibroblasts.

### Retinal progenitor cells (RPC)

Human retinal progenitor cells (hRPC), which can be expanded in the progenitor state and then differentiate into retinal cells before or after transplantation, represent an attractive solution for the production of a sufficient quantity of donor cells for therapeutic applications. They consist of a population of immature cells responsible for the production of retinal cells during embryonic development. The main source of acquisition is represented by ocular tissues of aborted fetuses 12 to 16 weeks of age, a time when retinal differentiation is well defined ^(46,47)^. hRPCs can be expanded for multiple passages in the undifferentiated state and can be induced to express photoreceptor markers (opsins) by means of *in vitro* differentiation or after subretinal implantation. The phase of hRPC expansion can be significantly extended under low oxygen conditions, generating a scalable cell source appropriate for generalized clinical applications^(47)^. hRPCs derived from fetuses have low immunogenicity ^(46)^ and have been used for subretinal or IVT injection (Table 1).

### Use of RPCs

Liu et al.^(46)^ used hRPCs in eight patients with RP, confirming the absence of tumor growths. The cells were injected into the subretinal space after vitrectomy, and the procedure has been proven safe, with an increase in the thickness of the outer nuclear layer detected by OCT. But, no significant changes in vision were observed, and the absence of improvement was explained by the fact that the patients were in a very advanced stage of the disease. In another study still underway, hRPCs derived from fetal tissues were administered by IVT injections under local anesthesia at different concentrations (0.1, 1, 2, and 3 million cells) in 28 patients with RP. So far (12 months of follow-up) the procedure has been proven safe (Clinical trial CT03073733)^(48)^. The cited study is coordinated by Dr. Barruch Kuppermann in partnership with the jCyte company and the California Institute for Regenerative Medicine (CIRM)^(49)^.

The availability of noninvasive high-resolution imaging techniques, the immunosuppressive nature of the subretinal space, and the existence of surgical techniques that permit transplantation provide a field favorable for studies in cell therapies for retinal diseases. Therapy using different stem cell sources has been proven safe and has the potential for functional cell rescue and/or replacements in experimental and clinical phase I/II studies.

Additional investigations will be necessary to identify the mechanisms that control the formation/disjunction of synapses (in order to improve the efficacy of the transplant with photoreceptor potential), factors that limit the survival of cells implanted into the subretinal space in areas of geographic atrophy (to improve the efficacy of the RPE implants), and factors that regulate the immunological vigilance of the subretinal space (to improve the long-term survival of cell transplants with photoreceptor ability and RPE).

Many of the tools needed to start reaching these objectives are currently available, and clinical studies have begun the journey aiming at the functional restoration of the retina, representing a new therapeutic arsenal in the fight against blindness.
